# Excision of whole intact mouse brain

**DOI:** 10.1016/j.mex.2023.102246

**Published:** 2023-06-08

**Authors:** David B. MacManus

**Affiliations:** a School of Mechanical & Materials Engineering, University College Dublin, Dublin, Ireland; b BRAIN Lab, School of Mechanical & Materials Engineering, University College Dublin, Dublin, Ireland

**Keywords:** Cranial bone, Dissection, Murine, Neural tissue, Mechanical characterization, Brain mechanics, Traumatic brain injury, Biomechanics, Material characterization, Material properties, Excision of intact mouse brain

## Abstract

Mechanical characterization experiments of brain tissue are performed to understand the mechanical behavior of brain tissue during normal physiology and pathophysiological processes including traumatic brain injury. Normal, healthy, undamaged, unfixed brain tissue specimens are required for these mechanical characterization experiments to ensure the properties being measured are not from damaged/diseased tissue which may lead to inaccurate and unreliable results regarding the mechanical behavior of healthy undamaged brain tissue. The process of excising brain tissue from the cranial vault of mouse cadavers can induce lacerations in the tissue that may affect its mechanical behavior. Therefore, it is imperative that brain tissue samples are excised without inducing damage to the tissue so that the normal undamaged mechanical properties can be measured. Here, a method to excise the entire intact mouse brain is presented:

• The scalp is resected exposing the anterior portion of the skull.
• Cranial bone is resected by incising along the cranial sutures and using the scalpel blade to remove the cranial segments.
• Connective tissue is resected and the brain is removed from the cranial vault.

## Graphical abstract

**Figure:**
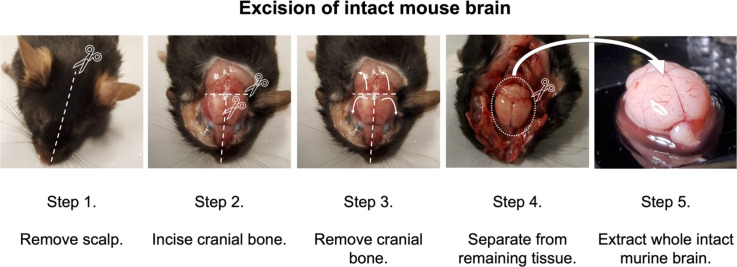

Specifications table

**Table:** 

Subject area:	Neuroscience
More specific subject area:	*Traumatic brain injury*
Name of your method:	*Excision of intact mouse brain*
Name and reference of original method:	*N/A*
Resource availability:	*N/A*

## Method details

### Background

The global incidence of traumatic brain injury (TBI) is on the rise and is estimated to effect 69 million people each year [1]. TBI is a mechanical injury by nature and has a spectrum of injury severity with an increasing likelihood of structural and functional damage to the brain as the injury severity graduates from mild to moderate, and severe [4]. Due to the mechanical nature of TBI, mechanical characterization of brain tissue is an increasingly common experiment performed to understand how the brain deforms under different loading scenarios and to develop models of TBI that can more accurately recapitulate the pathological deformation leading to TBI. Typically, prior to a TBI the brain tissue is assumed to be healthy and uninjured. Therefore, in order to capture biofidelic mechanical behavior of healthy uninjured brain tissue, mechanical characterization experiments should use samples from undamaged brains. Although human brain tissue properties are the gold standard for mechanical properties of brain tissue to study human physiology and disease, it is extremely difficult to source healthy human tissue. Therefore, laboratories usually opt to use an animal surrogate model for human tissue given the similarities between the mechanical properties of animal and human brain tissue [3]. Two common animal models for the mechanical characterization of brain tissue are the murine [3] and porcine models [2,3,5]. However, given the small size of the mouse brain, it is particularly challenging to excise whole intact mouse brains without damage. Further, to the best of the author's knowledge, there are no published articles or methods that detail the excision of whole intact mouse brain without damage. Therefore, a method to excise whole mouse brain without damaging the organ has been developed and is presented here.

### Required materials

Scalpel handle. Pointed tip scalpel blade. Nitrile gloves (double glove to reduce possibility of stick injury). Phosphate buffer solution or saline solution. Tweezers with rubber tips. Petri dish. Cutting board.

### Excision of intact mouse brain

**Fig. 1:**
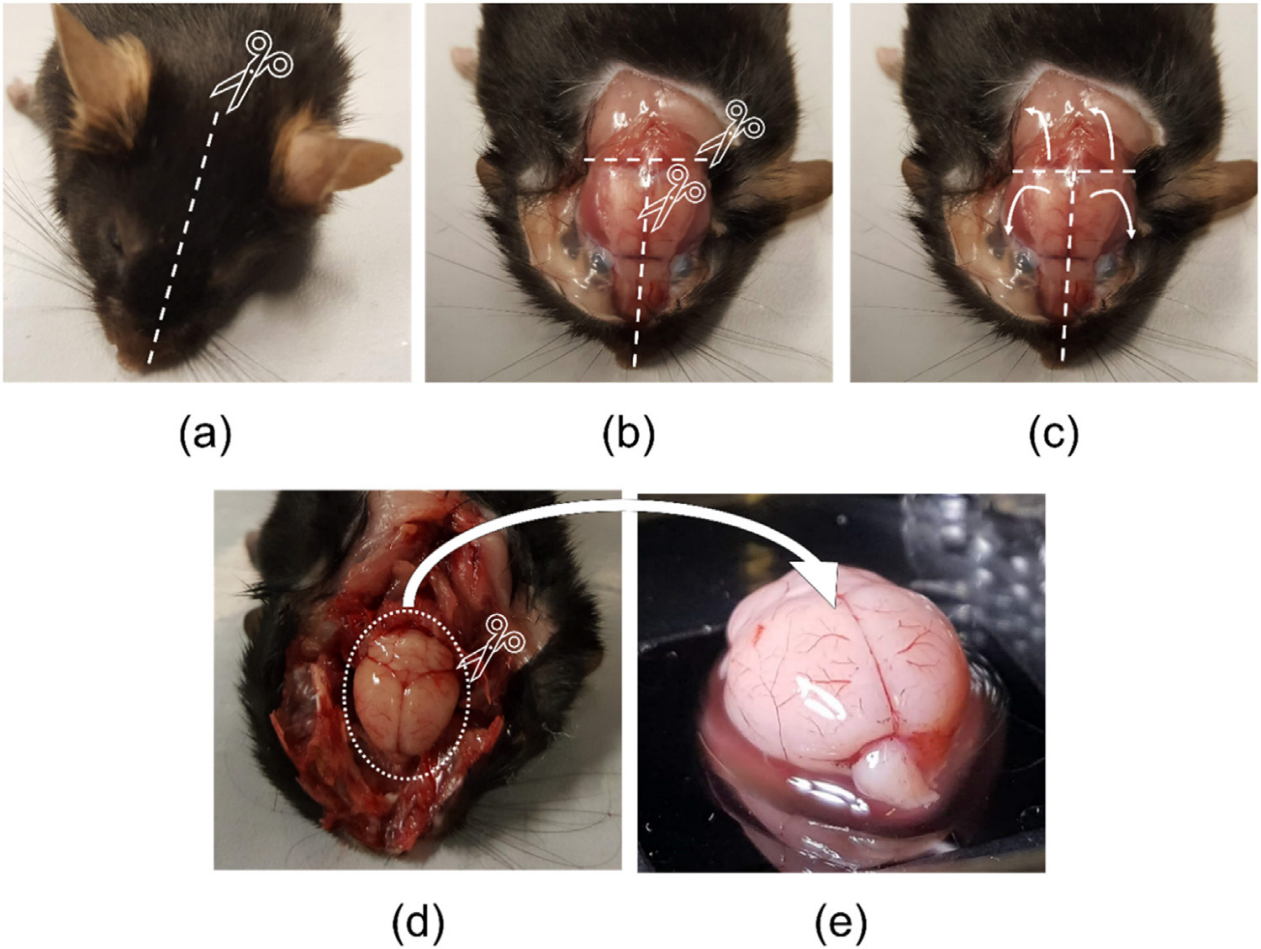
Outline of steps in the excision of intact mouse brain. (a) Make a sagittal incision along the centre of the scalp (white dashed line) using a handled surgical scalpel blade with a pointed tip. (b) Using the scalpel, incise along the sagittal suture of the cranial bone from the intersection of the sagittal and lambdoid sutures to the extremity of the nasal bone. Next, incise laterally across the lambdoid suture. (c) Use the scalpel blade and tweezers to peel back and resect the three segments of cranial bone in the direction of the white arrows exposing the underlying brain. (d) Use the scalpel blade to gently resect any connective tissue anchoring the brain to the lower skull cavity. (e) Use tweezers with rubber tips to gently remove the intact brain from the cranial vault.

**Fig. 2:**
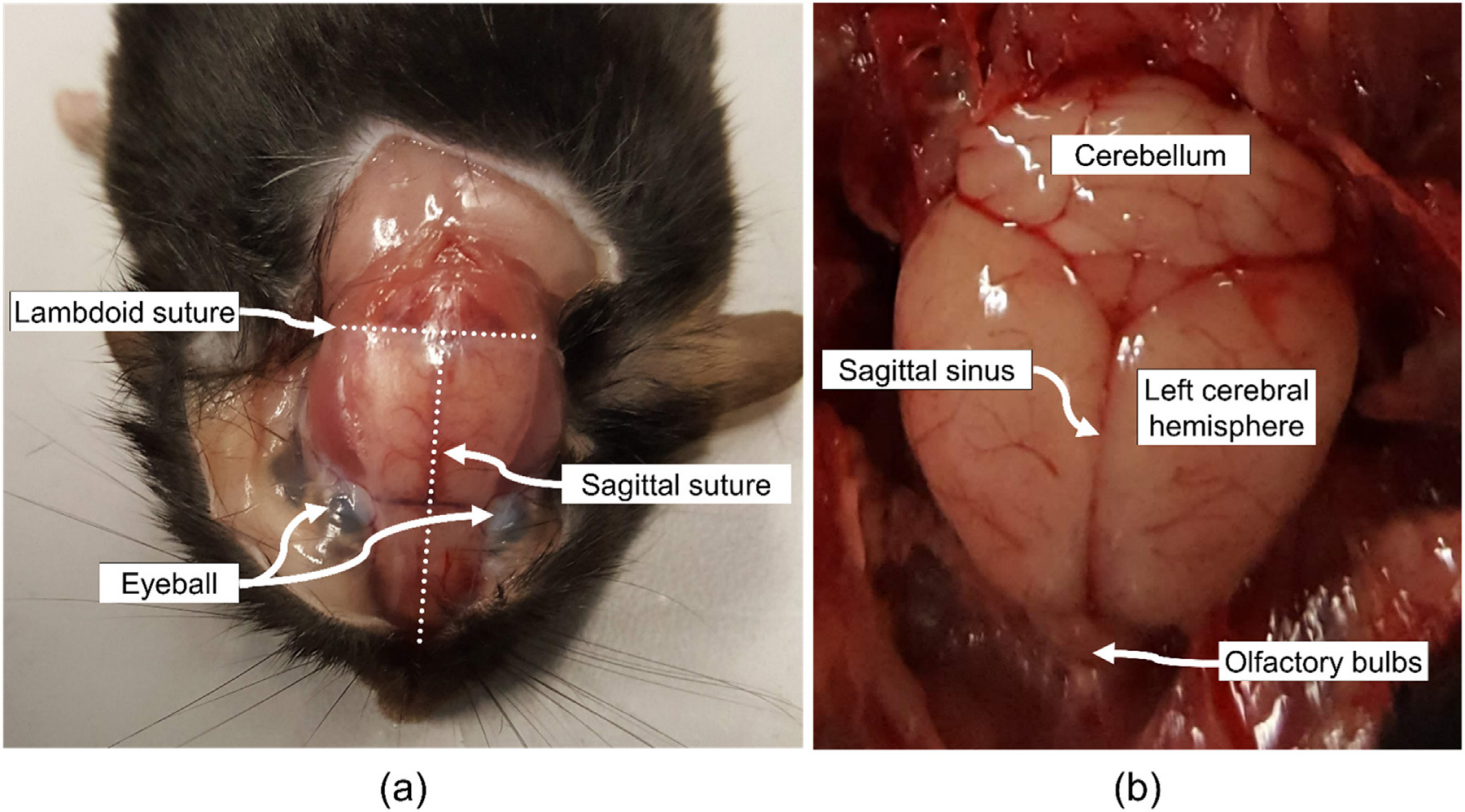
Major (a) cranial and (b) cerebral anatomical structures and landmarks for excision of mouse brain.

Using a pointed tip scalpel, create a sagittal incision along the centre of the scalp from the base of the neck to the tip of the snout and resect the scalp (Fig. 1a, white dashed line). If required, the incision can be extended further down the back to increase access to the skull. Wash scalpel blade in sterile phosphate buffer (PBS) or saline solution to remove any hair, blood, or debris. Fresh mouse cadaver cranial bone is relatively soft with an approximate thickness of 0.2 mm. These properties permit incising using a conventional surgical scalpel with a pointed tip. Therefore, continuing with the same scalpel that was used to resect the scalp, incise along the sagittal suture of the cranial bone from the intersection of the sagittal and lambdoid sutures (Fig. 2a) to the extremity of the nasal bone (Fig. 1b, white centre dashed line). Here, it is important to consider the average thickness of the mouse skull and to take care not to incise too deeply as to lacerate the underlying brain tissue. Note: The underlying sagittal sinus (Fig. 2b) of the cerebrum provides a small margin of error when incising through the cranial bone as this tissue is not in direct contact with the interior of the skull. Next, incise laterally across the lambdoid suture (Fig. 1b, horizontal white dashed line). Once both cranial bone incisions have been performed, use the scalpel blade and surgical tweezers to lift and resect each of the three segments of cranial bone in the direction of the white arrows exposing the underlying brain (Fig. 1c). Following resection of the cranial bone, use the scalpel blade to gently resect any connective tissue anchoring the brain to the lower skull cavity (Fig. 1d). Note: it may be more efficient to reposition the cadaver on its side or to invert the cadaver to aid access to the inferior connective tissues. Finally, using the tweezers, gently remove the intact brain from the cranial vault, wash the brain in a petri dish with fresh PBS or saline solution to remove any blood or other biological fluid and debris, and place into a petri dish and maintain hydration prior to testing (Fig. 1e). It is important to change scalpel blade after each excision to avoid sticking injuries as the blade will be blunted from incising the cranial bone. Note: wearing two pairs of nitrile gloves can also help prevent sticking injuries and is recommended when performing this method.

### Method validation

**Fig. 3:**
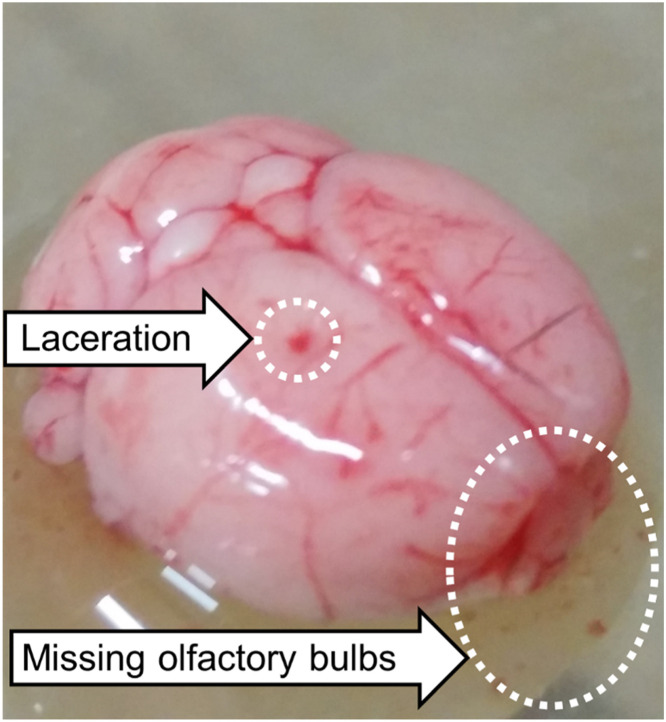
Common damage to mouse brain tissue during excision highlighting a laceration induced during the removal of the cranial bone segments and separation of the olfactory bulbs from the cerebrum.

Validation of this method is performed through visual inspection of the excised brain by examining the organ for any lacerations or damage that may have incurred during the excision process. If the brain has received any damage during the extraction process, then it should be discarded. Damage to the brain tissue is most likely to occur by lacerating the cerebral cortex when lifting away the cranial bone segments or through separation of the olfactory bulbs when removing the brain from the cranial cavity (Fig. 3). Visual inspection of the excised brain is a crucial step as it can often be difficult to observe lacerations due to the presence of vasculature.

## Ethics statements

This method was designed and developed exclusively using cadaveric mouse tissue and therefore is exempt from requiring ethical approval. All animal procedures were conducted in line with national legislation in accordance with European Commission Directive (2010/63/EU), as overseen by the Health Products Regulatory Authority of Ireland and the Animal Research Ethics committee of University College Dublin.

## CRediT authorship contribution statement

**David B. MacManus:** Conceptualization, Methodology, Writing – original draft, Writing – review & editing.

## Declaration of Competing Interest

The authors declare that they have no known competing financial interests or personal relationships that could have appeared to influence the work reported in this paper.
